# Exploration of helpful elements contributing to engage in physical activity in patients with cancer

**DOI:** 10.1007/s00520-024-08647-4

**Published:** 2024-07-11

**Authors:** Tomoko Matsui

**Affiliations:** Osaka University, Graduate School of Human Sciences, 1-2, Yamada-Oka, Suita, Osaka Japan

**Keywords:** Cancer, Patients with cancer, Physical activity, Moderate-or-higher-level intensity of physical activity, Health Action Process Approach

## Abstract

**Purpose:**

Although there are many reported benefits of physical activity for patients with cancer (e.g., improving quality of life), many patients with cancer do not meet the recommendations of physical activity guidelines. The amount of physical activity declines after diagnosis of cancer. This study aimed to clarify the elements that contribute engagement in regular moderate-or-higher-level intensity of physical activity (MHPA), based on the Health Action Process Approach (HAPA), among patients with cancer.

**Methods:**

Two web surveys were conducted among cancer outpatients, asking them to respond to a questionnaire. Participants provided information regarding their demographics, physical activity, purpose, advantages, disadvantages, barriers, triggers, and need for support for regular MHPA.

**Results:**

Categories were obtained for purposes, advantages, disadvantages, barriers, triggers, and support needs for regular MHPA. For example, the support they considered necessary to regularly engage in MHPA were developed 9 categories, such as *Improving the environment*, *Support for the implementation of PA (interpersonal)*, and *Reducing the burden*. Women and younger patients provided more statements about reducing their burden; younger patients also mentioned on improving the environment more among those who did not engage in MHPA. Additionally, patients with cancer who provided statements about interpersonal support reported higher barriers to physical activity.

**Conclusions:**

These findings will contribute to the development of a scale to measure the components of the HAPA on physical activity in patients with cancer. In addition, it will help develop a support system that promotes engagement in regular MHPA.

**Supplementary Information:**

The online version contains supplementary material available at 10.1007/s00520-024-08647-4.

## Introduction

Increasing attention has been paid to the promotion of physical activity (PA) in patients with cancer. Many benefits of PA have been reported in these patients. Examples include decreasing depression and stress levels [1–3], improving physical function and strengthening muscles [4], reducing the side effects of treatment [5], and maintaining or improving the quality of life [4, 6, 7]. A reduction in mortality has also been reported for some types of cancers [8]. Guidelines for PA in patients with cancer have also been reported, including recommended standards for PA [9–11]. Nevertheless, many patients with cancer do not meet the recommendations of PA guidelines [12, 13]. In addition, the amount of PA decreases after cancer diagnosis [14]. Therefore, several studies have been conducted to promote PA in patients with cancer.

According to one study [15], previous experience with PA influenced the amount of current level of PA. Therefore, especially for those who have not been physically active previously, it is not difficult to assume that regardless of how much they know that PA is good, it is difficult to implement. Studies that have focused on barriers to PA in patients with cancer have also found that during and after treatment patients with cancer have similar content regarding facilitators and discouragements related to PA as the general population, as well as content related to their cancer experiences [16, 17]. Factors contributing to promoting PA have been reported, including those related to the effects and benefits of PA and psychosocial aspects such as improving well-being and support [18, 19]. Common barriers to PA, such as bad weather, have been reported, as well as cancer and treatment-related barriers, such as fatigue and pain, and barriers related to beliefs about the safety of PA and fear of worsening symptoms [16, 18, 19].

Many studies have applied behavioral change theories, such as the theory of planned behavior [20], social cognitive theory [21], and the transtheoretical model (TTM) [22], to promote PA in patients with cancer. Theory-based behavioral interventions have been suggested as useful, but their effects are not large, depending on the theory [23, 24]. Studies that partially or completely utilize the Health Action Process Approach (HAPA) [25, 26], to promote PA in patients with cancer are being reported [3, 27–30]. The HAPA state that self-efficacy, outcome expectancy, and risk perception influence behavioral intentions, which promote health behaviors through planning [25, 26]. There is still a lack of knowledge in studies that especially examine the application of the HAPA to explain the engagement in PA among people with cancer [29, 30]. Additionally, in this model, resources, including social support, are considered to contribute to certain components such as behavior and intention. Although not a study on patients with cancer, one study using the HAPA reported that social support was positively associated with action planning, suggesting its importance in promoting PA [31]. Understanding what kind of support is needed for people with specific characteristics will give us a more helpful insight into developing interventions that are more acceptable to patients with cancer.

This study investigated barriers, advantages, disadvantages, purpose, triggers, and support needs of engaging in moderate-or-higher-level intensity of physical activity (MHPA) to extract the items that contribute to the components of the HAPA [25, 26] among patients with cancer. Then, the relationship between the content of the need and characteristics of the participants was examined to identify the characteristics of their support needs.

## Methodology

### Participants and procedure

Web surveys were conducted, and participants were asked to respond to a questionnaire through INTAGE HOLDINGS, Inc. The participants were patients with cancer who met the following criteria: 1) not currently hospitalized, 2) within five years of their cancer diagnosis, and 3) during or within six months of completing treatment for cancer. A screening survey was conducted to a panel that consisted of people who have received medication from a doctor for “cancer” within the last year. This survey for making the panel was conducted in advance and managed by INTAGE HOLDINGS, Inc.

Survey 1(S1): The former survey for making the panel was conducted in August 2019. The screening and main surveys were conducted in March 2020. Thirteen hundred and twenty-six members of this panel were asked to participate in the screening, of whom 577 participated. Of them, 311 met the screening criteria and responded to the survey.

Survey 2 (S2): The former survey for making the panel was conducted in August 2020. The screening and main surveys were conducted in January 2021. Fifteen hundred and eighty-eight members of this panel were asked to participate in the screening, of whom 1011 participated. Of them, 355 met the screening criteria and responded to the main survey.

### Measurements

#### Demographics (S1, S2)

Participants were asked about their age, sex, treatment information, time since initial diagnosis, and information on their disease. The responses obtained from a survey conducted in advance by the company were used to identify the types of cancer (S2). The participants subjectively responded to the Karnofsky Performance Scale (KPS) [32] from eight options (Table S1).

#### Engaging in PA (S1, S2)

The status of regularly engaging in MHPA was asked based on the TTM [22] and previous study [15]. The options are shown in Table S1. Regular PA means about three times a week for about 30 min each time.

#### Thoughts on engaging in regular MHPA (S1)

Participants were asked about the barriers, advantages, disadvantages, triggers, and purpose of regular MHPA. For barriers and advantages, we used each 10 items from the previous study [33] and asked them to select all ones that applied and to answer freely (barriers: “What are the reasons that you do not regularly engage in MHPA? Or, when you have difficulty engaging in MHPA, what are the reasons?”; advantages: “What do you think are the benefits of engaging in MHPA regularly?”). For the other components, respondents were asked to answer freely. Regarding disadvantages, participants were asked, “What do you think are the disadvantages of not regularly engaging in MHPA?” In the context of purpose, respondents were asked the following questions: “If you regularly engage in MHPA, what is your purpose for engaging in it?” and “For those who do not regularly engage in MHPA, if you do so, what is its purpose?” Regarding triggers, respondents who did not engage in MHPA regularly were asked, “What kind of triggers do you think would enable you to engage in regular MHPA?”.

#### Thoughts on engaging in regular MHPA (S2)

This consisted of 23 items, including those from Ishii et al. [33], and was considered specific to patients with cancer that were extracted based on S1. For each item, respondents were asked to choose from “1: I do not think so at all” to “5: I really think so.”

Those who did not engage in regular MHPA were asked about the support they required to engage in such PA. Implementors of such PA were asked about the necessary support for those who did not engage in the PA to do so.

### Analysis

Descriptive statistics were calculated. Qualitative data were analyzed with reference to content analysis. The author classified the responses, generated categories, and assigned category names. Two graduate students majoring in health psychology independently determined whether the category names and data contents were consistent. Items that did not match were discussed by the authors until they agreed. Other responses regarding barriers and advantages were analyzed by applying the responses to the items of the existing scale or the methods above. Additionally, a variable was created by assigning age to two groups: < 65 years and ≥ 65 years, which were assigned values of 0 and 1, respectively. For the sex variable, males were assigned 0 and females were assigned 1. For the developed categories on need, the author assigned a rating of 1 if the participants mentioned it and a rating of 0 if they did not. Using these binary variables, chi-square tests were performed to examine the associations with demographic variables using S2 data. *T*-tests were also conducted to examine the association between the support need and barrier items.

### Ethical considerations

This study was approved by the Ethics Committee of Waseda University (reference number: 2020–093). In both surveys, INTAGE HOLDINGS Inc. explained the purpose of the surveys on the web and participants were considered to have granted consent by responding.

## Results

### Participants and status of PA (S1) (Table S1)

A total of 301 effective responses were received. Regarding the time since the diagnosis of cancer, the most frequent response was “2 to less than 3 years” (*n* = 84, 27.9%). The most common cancer type was breast cancer (*n* = 150, 49.8%). Among those currently undergoing treatment or within six months of treatment, hormonal therapy was the most common (*n* = 185, 61.5%), followed by chemotherapy (*n* = 119, 39.5%). The responses to the KPS scale showed about 80% of all respondents answered better than to be able to carry on normal activities. A total of 125 respondents (41.5%) were regularly engaging in MHPA, but the most common answer was “I have not done it regularly since before I had cancer (*n* = 104, 34.6%).”

### Participants and status of PA (S2) (Table S1)

Data from 303 patients with cancer were analyzed. The most frequent response on the time since the diagnosis was “1 to less than 2 years” for 98 respondents (32.3%). The most common cancer type was breast cancer (*n* = 148, 48.8%). Hormonal therapy was the most common type of treatment currently undergoing or within 6 months after treatment, with 175 (57.8%) of the respondents, followed by chemotherapy (*n* = 124, 40.9%). Regarding the KPS scale, more than 80% of all respondents answered better than to be able to carry out normal activities. While the most common answer was “I have not done it regularly since before I had cancer (*n* = 107, 35.3%), 141 respondents (46.5%) were currently engaging in regular MHPA.

### Barriers for regular MHPA (Table 1)

**Table 1:** Barriers for regular MHPA (*N* = 301)

Categories	Examples of respondents	*n*	*%*
Boring to exercise	-	43	14.3
Not enough time	-	45	15.0
Not recommended by family members	-	6	2.0
Being slothful	-	86	28.6
Bad weather	-	26	8.6
Getting tired by exercise	-	116	38.5
Too much work	-	12	4.0
No one to exercise with	-	25	8.3
Lack of motivation	-	43	14.3
Lack of facilities	-	14	4.7
*Others*	-	46	15.3
Disease or disability	Due to high blood pressure and heart concerns	9	3.0
Pain	Because I still get sore scars	6	2.0
Decline in physical function	My hemoglobin level is low, and I am short of breath and cannot exercise	6	2.0
Not receiving permission from a doctor	My doctor has prohibited me	4	1.3
Side effects of treatment	Cannot do as much as I would like due to side effects of anticancer drugs	4	1.3
Poor physical condition	I do not feel well	3	1.0
Decline in physical strength	I have lost a lot of my physical strength	3	1.0
Being elderly	Old age and chronic illness	2	0.7
Not preferring higher intensity physical activity	Not like to do vigorous sports	2	0.7
Changing appearance	I would like to go to the gym but cannot go to take the shower	1	0.3
Lack of financial resources	Waste of money going to the gym	1	0.3
Social conditions	To prevent myself from getting COVID-19	1	0.3
Difficulty in continuing physical activity	Unable to continue	1	0.3
Nothing	Nothing is special	8	2.7

The common barriers included *Getting tired by exercise* (*n* = 116, 38.5%), *Being slothful* (*n* = 86, 28.6%), *Not enough time* (*n* = 45, 15.0%), *Lack of motivation* (*n* = 43, 14.3%), and *Boring to exercise* (*n* = 43, 14.3%). As a result of the classification of the contents of *Others* (*n* = 46, 15.3%), 14 categories were developed, such as *Disease or disability* (*n* = 9, 3.0%), *Pain* (*n* = 6, 2.0%), *Decline in physical function* (*n* = 6, 2.0%), *Not receiving permission from a doctor* (*n* = 4, 1.3%), and *Side effects of treatment* (*n* = 4, 1.3%).

### Advantages for regular MHPA (Table 2)

**Table 2:** Advantages for regular MHPA (*N* = 301)

Categories	*n*	*%*
Better health	169	56.1
Reduced stress and relaxed	161	53.5
Improved general endurance	152	50.5
Maintain an appropriate body weight	117	38.9
Fun and enjoyable	60	19.9
Improved appearance	40	13.3
Doable with friends	26	8.6
Challenging possibilities	26	8.6
Enhanced friendships	21	7.0
Received recognition from others for one' s abilities	6	2.0
*Others*	15	5.0
Preventing recurrence	1	0.3
Reducing swelling	1	0.3
Nothing/Not knowing	13	4.3

The common categories were as follows *Better health* (*n* = 169, 56.1%), *Reduced stress and relaxed* (*n* = 161, 53.5%), *Improved general endurance* (*n* = 152, 50.5%), and *Maintain an appropriate body weight* (*n* = 117, 38.9%). Other content relevant to the advantages were *Preventing recurrence* and *Reducing swelling* (respectively, *n* = 1, 0.3%).

### Disadvantages of not engaging in regular MHPA (Table 3)

**Table 3:** Disadvantages of not engaging in regular MHPA (*N* = 301)

Categories	Examples of respondents	n	%
***Worsening of cancer and side effects of treatment***		7	2.3
Increased risk of cancer progression or recurrence	Increased risk of recurrence	6	2.0
Unable to tolerate side effects	Physical weakness and inability to tolerate the side effects of anticancer drugs	1	0.3
***Deteriorating health***		94	31.2
Becoming overweight	Not being able to move around much because of illness and gaining fat in unwanted places	51	16.9
Developing physical problems, becoming unhealthy	Physical complaints	20	6.6
Getting tired more easily	Physical weakness and increased fatigue	10	3.3
Increasing risk of disease	It is a risk for all adult diseases	12	4.0
Accelerated aging and loss of longevity	Not be able to live longer	6	2.0
***Decline in physical strength and physical function***		140	46.5
Declining in physical strength	Physical strength declines	66	21.9
Loss of muscle strength	Muscle wasting	52	17.3
Decline in physical functions	Decrease in physical ability and weakness of the body	29	9.6
Weakening of bones	Loss of muscle strength and bone mass	3	1.0
Increasing difficulty in controlling bowel movements	Constipation	2	0.7
Decreased metabolism	Metabolism does not improve	2	0.7
Increased risk of injury	Physical mobility is weakened, increasing the risk of injury	1	0.3
Becoming poor posture	Metabolism and posture get even worse	1	0.3
***Decreased amount of physical activity and stagnation of social activities***		12	4.0
Being lack of physical activity	More and more, I stop moving	5	1.7
More likely to stay one' s house	If I stay house inside, that will become the norm	3	1.0
Decreased interaction with others	Relationships with friends become estranged	3	1.0
Disturbed rhythm of lifestyle	The rhythm of my lifestyle is disrupted	2	0.7
***Worsening of mental health***		21	7.0
Diminishing motivation and worsening mental health	I think I will fall into negative and backward thinking mentally	10	3.3
Increasing stress	I will not be able to relieve stress	11	3.7
***Nothing***		54	17.9
None in particular	There is none in particular	36	12.0
Not knowing	I do not know	18	6.0

Responses were obtained for 356 items, and were classified into six categories: *Worsening of cancer and side effects of treatment* (*n* = 7, 2.3%), *Deteriorating health* (*n* = 94, 31.2%), *Decline in physical strength and physical function* (*n* = 140, 46.5%), *Decreased amount of physical activity and stagnation of social activities* (*n* = 12, 4.0%), *Worsening of mental health* (*n* = 21, 7.0%), and *Nothing* (*n* = 54, 17.9%).

### Purpose of regular MHPA (Table 4)

**Table 4:** Purpose of regularly engaging in MHPA among patients with cancer (*N* = 301)

Categories	Examples of respondents	*n*	%
***Preventing cancer recurrence or worsening***		7	2.3
Preventing and improving cancer	If I do not exercise, my cancer might relapse	7	2.3
***Staying healthy***		84	27.9
Maintaining and improving health	For a healthy body	41	13.6
Dieting and maintaining body shape	For health and maintaining body weight	36	12.0
Maintaining and improving immunity	To enhance the immune system	6	2.0
Preventing and improving lifestyle diseases	For preventing lifestyle-related diseases	5	1.7
Living longer and preventing aging	To live longer	5	1.7
***Maintaining and improving physical strength and physical function***		100	33.2
Restoring, maintaining, and improving physical strength	To maintain physical strength	55	18.3
Maintaining and strengthening muscle strength	To prevent muscle loss	25	8.3
Maintaining and improving physical functions	Improving the ability to walk	17	5.6
Rehabilitation	For rehabilitation of the left arm, which became difficult to move immediately after the surgery, and for stress relief	3	1.0
Strengthening of bones	Minimizing loss of bone density	2	0.7
Improves posture	For postural correction	2	0.7
***Preventing or improving mental health problems and preventing cognitive decline***		25	8.3
Distraction, relaxation, stress reduction	Relieving psychological stress and refreshing the mind	17	5.6
Enjoyment	For enjoyment	5	1.7
Self-satisfaction	For self-satisfaction	1	0.3
Reducing anxiety	I am anxious if I do engage in physical activity	1	0.3
Preventing cognitive decline	Preventing dementia by going to the gym	1	0.3
***Engaging in physical activity, hobbies, and social activities***		61	20.3
Walking	I like to walk. I want to walk	18	6.0
Habits of physical activity	Because it's a habit	10	3.3
Going shopping	Walking in the store for shopping, etc	5	1.7
Working, returning to the working, and doing household	Accompanying my children to school	4	1.3
Attend gym/fitness club	Because I like to work out at the gym	4	1.3
Engaging in volunteer activities	Volunteer work at club for the elderly to watch over children, clean up parks, etc	3	1.0
Playing golf	Practicing golf	3	1.0
Getting the exercise required	For getting more exercise	2	0.7
Climbing / hiking	Hiking	2	0.7
Riding a bicycle	Bicycle	2	0.7
Doing yoga	Yoga	2	0.7
Engaging in radio exercises	Attending radio exercises	2	0.7
Swimming and walking in water	Walking in the pool	2	0.7
Engage in hobbies	Hobbies	2	0.7
Interacting with others	I do it to maintain my health, fitness, and shape but also to refresh my mind through conversation with my friends	2	0.7
Going skiing	I had enjoyed skiing until now, so I would like to maintain my basic physical strength so that I can continue to ski in the future	1	0.3
Doing hula dance	Hula dance	1	0.3
Running	Running	1	0.3
Others	I only wish I could move my body without a slight fever	1	0.3
***Others***		2	0.7
Reducing medical expenses	Reducing medical costs. If I am not healthy, I have to pay for medical expenses	1	0.3
Others	Outcome/Harvest	1	0.3
***Nothing***		27	9.0
None in particular	Not especially	27	9.0

Three hundred and thirty responses were obtained and classified into seven categories: *Preventing cancer recurrence or worsening* (*n* = 7, 2.3%), *Staying healthy* (*n* = 84, 27.9%), *Maintaining and improving physical strength and physical function* (*n* = 100, 33.2%), *Preventing or improving mental health problems and preventing cognitive decline* (*n* = 25, 8.3%), *Engaging in physical activity, hobbies, and social activities* (*n* = 61, 20.3%), *Others* (*n* = 2, 0.7%), and *Nothing* (*n* = 27, 9.0%).

### Triggers for engaging in regular MHPA (Table 5)

**Table 5:** Triggers for engaging in regular MHPA (*N* = 176)

Categories	Examples of respondents	*n*	*%*
***Improved living environment and lifestyle***		20	11.4
Reduced work and household chores, more time available	Reducing the amount of childcare, caregiving, and household chores	10	5.7
Improved environment for physical activity	If there is a facility nearby	7	4.0
Removed financial burden/financial affordability	If I can afford it in terms of time and finances	5	2.8
***Improved cancer treatment and physical conditions***		44	25.0
Getting in better physical condition	Physical condition should recover and stabilize	13	7.4
Increased muscle strength and stamina	I think I can do it once I have a little more muscle strength	8	4.5
Reduced side effects	I think I can start when the side effects of the anticancer drugs are resolved	7	4.0
Reduced pain	When physical pain is relieved than now	7	4.0
Finished treatment	I would like to do it if the treatment is finished and progress is good	5	2.8
Obtained a doctor's permission/assurance	I would like to do this as soon as my doctor gives me permission	5	2.8
Relieved breathlessness	I want to exercise when the breathlessness and cancer pain is gone	3	1.7
Relieved fatigue	If the side effects are gone and no fatigue	2	1.1
Others	When my hormone treatment is finished and my hair is back to normal, I can take a bath at the gym	1	0.6
***Recognize worsening physical condition***		12	6.8
Worsening of physical condition	If the blood examination results are worse	6	3.4
Increasing/decreasing from the appropriate weight	When I am overweight due to hormonal treatment	6	3.4
***Understanding the positive changes from engaging in MHPA***		6	3.4
Learning about the benefits of MHPA	It can be effective in the fight against cancer	5	2.8
Rewards	If there is something to look forward to after that	1	0.6
***Increase motivation for MHPA***		11	6.3
Increasing motivation	Raising motivation	7	4.0
Having the will to do	I think it should do it voluntarily, rather than as a trigger	4	2.3
***Obtaining support from others***		26	14.8
Being invited by someone/doing with someone	I might do it if someone invited me to do it	17	9.7
Receiving recommendations/encouragement from others	My family doctor tells me (to do it)	9	5.1
***Start with the easy steps***		3	1.7
Doing what can be easily	Easy exercise	3	1.7
***Improved weather and social conditions***		10	5.7
Improved climate	If there is a warm and pleasant day	6	3.4
Calming down of the social impact of the Covid-19	Once the weather improves and the COVID-19 is over, I plan to attend a city facility	5	2.8
***Other***		1	0.6
Problems solved	When my problems are solved	1	0.6
***Nothing***		54	30.7
Nothing in special	None	30	17.0
Not knowing	I do not know	13	7.4
Do not want to or w will not engaging in MHPA	I do not do	6	3.4
Do not feel the need of MHPA	I do not need it right now	3	1.7
Not being able to do	I cannot because I am handicapped	2	1.1

The 199 responses were classified into ten categories: *Improved living environment and lifestyle* (*n* = 20, 11.4%), *Improved cancer treatment and physical conditions* (*n* = 44, 25.0%), *Recognize worsening physical condition* (*n* = 12, 6.8%), *Understanding the positive changes from engaging in MHPA* (*n* = 6, 3.4%), *Increase motivation for MHPA* (*n* = 11, 6.3%), *Obtaining support from others* (*n* = 26, 14.8%), *Start with the easy steps* (*n* = 3, 1.7%), *Improved weather and social conditions* (*n* = 10, 5.7%), *Other* (*n* = 1, 0.6%), and *Nothing* (*n* = 54, 30.7%).

### Support needed to engage in regular MHPA (Table 6)

**Table 6:** Support needed to engage in MHPA (*N* = 303)

Not implemented(*n* = 162)				Implementers (*n* = 141)			
Categories	Examples of respondents	*n*	*%*	Categories	Examples of respondents	*n*	*%*
*Motivational support*		13	8.0	***Motivational support***		30	21.3
Support for one's own intentions, thoughts, and feelings	I think it depends on my motivation	11	6.8	Support for one's own intentions, thoughts, and feelings	If they have the will to do it themselves, they can do it	19	13.5
Raising awareness of the need for MHPA	If I feel it is necessary	1	0.6	Setting a goal	Specific goals	2	1.4
Providing a trigger	If there is a trigger	1	0.6	Raising awareness of the need for MHPA	Being strongly aware that it is for oneself	3	2.1
*Reducing the burden*		26	16.0	Providing a trigger	Motivation and trigger	1	0.7
Financial support	Financial assistance	15	9.3	Providing information on PA	Advertising and distributing guidelines for prevention and measures, such as how much exercise to do per day and what happens, and the risk of what happens if you do not work out	5	3.5
Support for daily life	If there is someone who can do housework and childcare for me, I would like to do it	5	3.1	Recommendations from others	The government or a doctor will recommend the implementation of exercise	3	2.1
Support for those who cannot take time	Time availability	8	4.9	***Reducing the burden***		15	10.6
*Improving the environment*		25	15.4	Financial support	I think it would be possible if there was a facility or other means of exercise that would be less of a burden financially	11	7.8
Providing opportunities for MHPA	If there are groups or something, I will be able to enjoy it	2	1.2	Support for daily life	I am busy with the time required to take care of my family, so if I could be freed from this burden, I would be able to do it	1	0.7
Arranging facilities and environment for MHPA	I would like to do it, but I cannot find such a facility	10	6.2	Support for those who cannot take time	Being able to afford the time	4	2.8
Arranging an environment for engaging in mandatory MHPA	If there is an environment in which I have to do it, I can continue to do it	2	1.2	***Improving the environment***		24	17.0
Preparing an environment dedicated to cancer patients	Classes for breast cancer patients	2	1.2	Providing opportunities for MHPA	Develop a system that makes it easy to join something like a group	4	2.8
Preparing an environment where people do not have to worry about the presence of others	If I no longer care about my appearance	2	1.2	Arranging facilities and environment for MHPA	It would be nice to have a facility where people can easily start exercising	11	7.8
Providing services that can be used in varying situations	Gym tickets available only once a week	1	0.6	Improving access to facilities for engaging in MHPA	I often wish there were facilities for exercise in the hospital where I am treated	8	5.7
Enabling environment for online implementation	I can have one-on-one online lessons	2	1.2	Preventing infectious diseases	Prevention of COVID-19	1	0.7
Preparing an environment for beginners	An environment that is easy to conduct even for newcomers	1	0.6	***Support for the implementation of PA (non-interpersonal)***		20	14.2
Improving access to facilities for engaging in MHPA	I might be able to go if I had shuttle service	4	2.5	Providing teaching materials	Providing examples just like in a textbook	2	1.4
Preventing infectious diseases	Solid infection prevention measures	2	1.2	Purchasing or lending exercise equipment	Lending of exercise equipment	2	1.4
*Support for the implementation of PA (non-interpersonal)*		6	3.7	Adding an element of fun and enjoyment	It could be fun if there is an element of fun, such as an app that adds points to the score	7	5.0
Providing teaching materials	An easy manual	3	1.9	Making the purpose different from PA	Trying to have other objectives than just walking, such as going to see the scenery, buying something, using a smartphone app, etc	2	1.4
Purchasing or lending exercise equipment and exercise support systems appropriate to one' s needs	There should be some kind of health advisor	2	1.2	Utilizing apps and games	I feel a sense of accomplishment if I have a game, app, or something that gives me immediate results	6	4.3
Preparing coping strategies in case something happens	Being ready to deal with things immediately	1	0.6	Support to engage in small steps	Just start by taking a little longer than usual to do one' s usual shopping	5	3.5
Support to engage in small steps	It would be good to be able to do it while doing something, such as watching TV	1	0.6	***Support for the implementation of PA (interpersonal)***		38	27.0
*Support for the implementation of PA (interpersonal)*		27	16.7	Support to gain understanding from family and friends	Understanding and support from family and friends	1	0.7
Support to gain understanding from family and friends	My wife is a very anxious person and does not want me to do it hardly, so if she understands of that	1	0.6	Engaging in MHPA with others	Support from people whom engage in MHPA with	25	17.7
Engaging in MHPA with others	Maybe I can do it if someone engages in PA with me	11	6.8	Receiving instruction and support from professionals	If there is a cheap or free gym that has trainers within a three minute walk, I might go there	2	1.4
Receiving instruction and support from professionals	If I could get a personal, medically knowledgeable trainer to instruct me at a low cost	10	6.2	Implementing PA under medical supervision	Management by a doctor	2	1.4
Receiving personalized instruction and advice	Something that people could input their exercise status and get personalized advice	3	1.9	Receiving assistance	Use of day care services and home-visit nursing care services	3	2.1
Receiving care	I can do it with the help of helpers and visiting nurses	4	2.5	Receiving personalized instruction and advice	First, support for each individual's different symptoms	1	0.7
Receiving mental health care	Maybe mental health care would help	1	0.6	Receiving advice from others in the same situation	Getting advice from people in the same condition	1	0.7
*Evaluating the implementation of PA*		2	1.2	Receiving encouragement	Encouragement	4	2.8
Preparing a reward	If there is a point system and we can get something or use a service, we can continue to do it	2	1.2	Others	Support from others for the exercise itself	2	1.4
*Support for improvement of physical condition*		14	8.6	***Evaluating the implementation of PA***		7	5.0
Obtaining a doctor's permission	If my doctor gives me permission, I can do it	1	0.6	Preparing a reward	An application that allows ones to earn points based on their activities	3	2.1
Managing pain and side effects	When the side effects of the medication are gone. If the days when I cannot go out for more than a week in a row due to side effects are gone	9	5.6	Aid in recognizing the effectiveness of PA	It would be nice to realize the benefits of doing	1	0.7
Support for recovery of physical strength and condition	I cannot do it with my current physical strength. I would like to do it after getting stronger through light physical activities	4	2.5	Keeping a record	Keep records and compare	2	1.4
*Difficulties due to social situations and physical conditions*		20	12.3	Having the achievements evaluated	Actually, have someone look at one' s results	1	0.7
End of the COVID-19	I would like to do this when the COVID-19 related crisis calms down	7	4.3	***Support for improvement of physical condition***		1	0.7
Difficulties due to illness or treatment	I also have a heart disease so I cannot do it even if I had assistance	9	5.6	Support for recovery of physical strength and condition	Depends more on physical condition than on support	1	0.7
Difficulties due to old age	I am too old to walk fast. I would like to move up to a moderate level if I can do light intensity with no difficulty	1	0.6	***Difficulties due to social situations and physical conditions***		5	3.5
Others	I cannot do it	2	1.2	End of the COVID-19	If the COVID-19 disaster converges, one's mind will change	3	2.1
*None*		50	30.9	Difficulties due to illness or treatment	People who need assistance cannot do it	1	0.7
No need for support	Not engaging in physical activity even with support	11	6.8	Difficulties due to old age	I think it is fine as it is now, with age-appropriate exercise	1	0.7
Not willing to answer	I do not want to answer	1	0.6	***None***		25	17.7
None in particular/not sure	I cannot think of anything in particular	38	23.5	Support to make PA a habit	Because it has not become a habit	1	0.7
				No need for support	No support needed	3	2.1
				None in particular/not sure	I cannot think of any	21	14.9

Among those who were not engaging in regular MHPA (*n* = 162, 53.5%), 192 elements were extracted from the responses, yielding 37 subcategories. Among those who were engaging in regular MHPA (*n* = 141, 46.5%), 179 elements were extracted, resulting in 39 subcategories. Finally, nine categories were developed regarding the support need: *Motivational support*, *Reducing the burden*, *Improving the environment*, *Support for the implementation of PA (non-interpersonal)*, *Support for the implementation of PA (interpersonal), Evaluating the implementation of PA*, *Support for improvement of physical condition*, *Difficulties due to social situations and physical conditions, and None*.

For those who were not engaging in regular MHPA, *Support for the implementation of PA (interpersonal)* (*n* = 27, 16.7%), *Reducing the burden* (*n* = 26, 16.0%), *Improving the environment* (*n* = 25, 15.4%) were commonly mentioned. For those who were engaging in regular MHPA, *Support for the implementation of PA (interpersonal)* (*n* = 38, 27.0%) was most reported, followed *Motivational support* (*n* = 30, 21.3%), and *Improving the environment* (*n* = 24, 17.0%).

### Relationships between support need categories and demographic and barriers variables

We conducted chi-square tests and *t*-tests using the support need categories and variables of barriers and demographics (age and sex) among those who did not engage in such PA (*n* =162) (Table S2, S3). There is a significant association between sex and mentions of support need, female patients provided more statements about reducing their burden(*χ*^*2*^ = 6.310, *df* = 1, *p* = .012, *φ*= .197). Significant associations were found between age and mentions of support need, younger participants provided more statements about reducing their burden (*χ*^*2*^ = 4.592, *df* = 1, *p* = .032, *φ*= -.168), and about improving the environment (*χ*^*2*^ = 4.154, *df* = 1, *p* = .042, *φ*= -.160). Patients with cancer who provided a statement about interpersonal support for the implementation of PA showed higher barriers to regular MHPA (*t* (160) = -2.819, *p* = .005, *d* = -0.594).

## Discussion

This study investigated and clarified the thoughts or support needed to engage in regular MHPA among patients with cancer, based on the HAPA model [25, 26].

### Status of PA among patients with cancer

 Approximately 80% of the participants in this study (S1, S2) had some symptoms but little difficulty in performing daily activities, it is assumed that most of them were capable of engaging in MHPA. However, less than half of the participants engaged in regular MHPA. More than 70% of those who currently engaged in regular MHPA had been doing so since before they developed cancer (S1, S2), suggesting the importance of considering past experiences, similar to a previous study [15]. 

### Contents of thoughts regarding MHPA

To understand the components of the HAPA model [25, 26], respondents were asked about the following items: barriers and advantages of regular MHPA, disadvantages of not engaging in it, purpose and triggers for it, and support needs for it. As a result, as in previous studies [16–19], both general and cancer-related contents were reported in this study. Participants more reported the general items presented based on previous studies [33]; for instance, *Being slothful* (28.6%) and *Not enough time* (15.0%) in barriers; most of contents in advantages; *Decline in physical strength and physical function* in disadvantages of not engaging (46.5%); and *Staying healthy* (27.9%) in purpose. For some questions, items that could be considered cancer specific were reported, although the number of reports was small. For example, *Changing appearance* (0.3%) *in barriers; Preventing recurren*ce and *Reducing swelling* (0.3%) in advantages; *Cancer-specific information on cancer recurrence and side effects* (2.3%) in disadvantages of not engaging; and *Preventing cancer recurrence or worsening* (2.3%) in purpose. Information about benefits that are specific to patients with cancer, such as the reduction of side effects of treatment [5] and mortality in some types of cancer [8], may not have been prevalent. At the time of the survey, there were no PA guidelines for Japanese patients with cancer. Additionally, even if the respondents intended to answer as cancer-related or specific to patients with cancer, it is possible that such respondents were encompassed by the word “health.” Fewer reports have been made; however, these detailed results, which contain the remarks of the participants, would be valuable resources. As for the triggers for engaging in regular MHPA*,* categories such as *Understanding the positive changes from engaging in MHPA*, which constitute the HAPA model [25, 26] were obtained. Additionally, the participants mentioned hopeful situational improvements as a trigger. In the case of patients with cancer, such improvements sometimes may not be possible. Thus, it may also be important to increase self-efficacy and support that they need. Meanwhile, the importance of planning for regular MHPA implementation was rarely perceived among them.

The HAPA [25, 26] is considered beneficial for explaining the promotion of PA among people with cancer, but there are insufficient reports on its applicability [3, 27–30]. In the future, for example, by utilizing the content obtained in this study, psychological scales that measure the components of the HAPA model [25, 26] specific to Japanese people with cancer and examining the fit of the model to them can be developed. This contributes to gaining a more accurate understanding of and promoting the physical activity practices.

### Support needed to engage in regular MHPA

Nine categories were developed in this study. The categories in which the most frequent comments were provided differed between those who were currently engaged in regular MHPA and those who were not. Those who implemented regular MHPA tended to focus on motivation, while those who did not appear to focus on behavior, with relatively more statements related to interpersonal support and burden reduction. In considering support measures, it is important not only to intervene with individuals, but also to adjust the environment and reduce their daily burdens. For example, setting up an intervention program that utilizes the strength of peers, as reported in previous studies [34], may satisfy the need of *Improving the environment* and *Support for the implementation of PA (interpersonal)*.

Additionally, the last two categories, *Difficulties due to social situations and physical conditions* and *None*, can hardly be called “support.” It is assumed that these results were obtained because this study asked about support-targeting engagement in the MHPA. Engaging in regular MHPA have often been recommended and studied in this field. However, in recent years, the effectiveness of light-intensity physical activity (LPA) has attracted attention because of the complex physical conditions of patients with cancer, fatigue, and the low rate of PA [35]. For example, LPA is positively associated with depressive symptoms, physical function, and quality of life [2, 36]. For those who have made statements in these categories, it may be acceptable to encourage LPA.

### Relationship between support needs and individual characteristics

This study showed that patients with cancer who provided a statement about interpersonal support for the implementation of PA reported higher barrier levels to regular MHPA, female and younger patients provided more statements about reducing their burden, and younger patients also more mentioned improving the environment. Given that support needs differ depending on the participants’ characteristics, it is necessary to take these into account when considering support strategies.

Additionally, the implementation of PA may be addressed as a problem-solving therapy for patients with cancer [37]. In such cases, the list of support needs obtained in this study can be used to develop a solution, and the results of this study have the potential for use in clinical situations. Furthermore, a healthcare provider’s recommendation was associated with higher levels of PA among cancer survivors [38]. The results of this study may be utilized by healthcare providers to recommend PA to their patients.

### Limitation

This study had several limitations. First, it was conducted as an online survey; therefore, there may have been a selectivity bias. Therefore, caution should be exercised when generalizing this study. Second, some components, such as support needs, were answered in an open-ended format; therefore, it is possible that items that were not brought to the participants' attention were not captured. Furthermore, as this study was conducted during the COVID-19 pandemic, its impact should be considered. For example, in the category of difficulties, the difficulty of not being able to engage in PA until the COVID-19 outbreak was mentioned. Despite these limitations, it is useful to provide a comprehensive description of thoughts on support strategies and other components for patients with cancer to engage in PA, especially MHPA.

## Conclusion

This study enabled us to obtain a comprehensive understanding of the thoughts of patients with cancer on engaging in regular MHPA. Developing a scale for measuring the HAPA components for patients with cancer in terms of psychology and examining the application of the HAPA [25, 26] to explain engagement in the MHPA is expected.

This study suggests that it is necessary to devise support strategies tailored to participants’ characteristics. It is also important not only to intervene with individuals but also to adjust the environment. It is expected that our results will be utilized, and that interventions or support will be provided by local governments and medical institutions in the future.

### Supplementary Information

Below is the link to the electronic supplementary material.

Supplementary file1 (PDF 215 KB)

## Data Availability

Data cannot be shared openly but are available on request from author.
