# Supplementary material for: Exploration of helpful elements contributing to engage in physical activity in patients with cancer
Source: Support Care Cancer. 2024 Jul 11;32(8):505. doi: 10.1007/s00520-024-08647-4 (PMC11239716; doi:10.1007/s00520-024-08647-4)
Supplement: Supplementary file 1 — Supplementary file1 (PDF 215 KB) [file 520_2024_8647_MOESM1_ESM.pdf]

Table S1 Demographic information

	Survey 1 (*n* = 301)		Survey 2 (*n* = 303)	
	Mean	*SD*	*Mean*	*SD*
Age	60.0	11.6	59.3	12.1
	n	*%*	*n*	*%*
Sex				
Male	126	41.9	124	40.9
Female	175	58.1	179	59.1
Duration since diagnosis (months)				
Less than 1 year	36	12.0	44	14.5
1-2years	67	22.3	98	32.3
2-3years	84	27.9	65	21.5
3-4years	58	19.3	47	15.5
4-5years	56	18.6	49	16.2
Cancer types				
Breast	150	49.8	148	48.8
Prostate	50	16.6	41	13.5
Lung	26	8.6	24	7.9
Colon	25	8.3	18	5.9
Stomach	14	4.7	11	3.6
Blood	10	3.3	8	2.6
Ovarian	2	0.7	4	1.3
Kidney	1	0.3	3	1.0
Pancreas	10	3.3	-	-
Liver	8	2.7	-	-
Thyroid	5	1.7	-	-
Cervical	4	1.3	-	-
Uterine body	3	1.0	-	-
Bladder	3	1.0	-	-
Esophagus	3	1.0	-	-
Brain	1	0.3	-	-
Others	17	5.6	52	17.2
Treatment ststus (undergoing or within 6 months after treatment)				
Surgery	97	32.2	92	30.4
Chemotherapy	119	39.5	124	40.9
Radiation therapy	25	8.3	42	13.9
Hormone therapy	185	61.5	175	57.8
Others	67	22.3	59	19.5
Karnofsky Performance Status				
Normal, no complaints	128	42.5	121	39.9
Able to carry on normal activities. Minor signs or symptoms of disease	108	35.9	128	42.2
Normal activity with effort	46	15.3	39	12.9
Care for self. Unable to carry on normal activity or to do active work	13	4.3	13	4.3
Requires occasional assistance, but able to care for most of his needs	4	1.3	1	0.3
Requires considerable assistance	2	0.7	0	0.0
Disabled, requires special care and assistance	0	0.0	1	0.3
Very sick, hospitalization is necessary	0	0.0	0	0.0
Status of regular MHPA				
I was doing it regularly before I had cancer and continue to do it now	94	31.2	99	32.7
I used to do it regularly before I had cancer, but not since I had cancer	61	20.3	44	14.5
I have not done it regularly since before I had cancer	104	34.6	107	35.3
I have been doing it regularly since I had cancer	31	10.3	42	13.9
I used to do it regularly since I had cancer, but I do not do it anymore	11	3.7	11	3.6

Table S2 Relationships between support need categories and demographic variables among participants those who were not implemented（*n* =162)

	Males (*n* = 53)		Females (*n* =109)		$\chi 2$	*P/Fisher's exact test*		φ	Younger people (*n* = 115)		Older adults (*n* = 47)		$\chi 2$	*P/Fisher's exact test*		φ
	n	*%*	*n*	*%*					*n*	*%*	*n*	*%*				
Motivational support	4	7.55	9	8.26	-	1.000	a	.012	9	7.83	4	8.51	-	1.000	a	.011
Reducing the burden	3	5.66	23	21.10	6.310	.012	*	.197	23	20.00	3	6.38	4.592	.032	*	-.168
Improving the environment	5	9.43	20	18.35	2.172	.141		.116	22	19.13	3	6.38	4.154	.042	*	-.160
Support for the implementation of PA (non-interpersonal)	0	0	6	5.50	-	.179		.137	6	5.22	0	0	-	.182	a	-.125
Support for the implementation of PA (interpersonal)	6	11.32	21	19.27	1.621	.203		.100	21	18.26	6	12.77	0.725	.394		-.067
Evaluating the implementation of PA	0	0	2	1.83	-	1.000	a	.078	2	1.74	0	0	-	1.000	a	-.071
Support for improvement of physical condition	5	9.43	9	8.26	-	.774	a	-.020	8	6.96	6	12.77	-	.234	a	.094
Difficulties due to social situations and physical conditions	10	18.87	10	9.17	3.096	.078		-.138	11	9.57	9	19.15	2.832	.092		.132
None	23	43.40	27	24.77	5.798	.016	*	-.189	32	27.83	18	38.30	1.715	.190		.103

a) Fisher's exact test

* p < .05

Table S3 Relationships between support need categories and barrier of MHPA among participants those who were not implemented （*n* =162)

	Described		Not described		Described		Not described		*t*	*P*		*Cohen's d*
	n	%	*n*	%	*M*	*SD*	*M*	*SD*				
Motivational support	13	8.02	149	91.98	61.92	13.41	65.76	14.01	0.949	.344		.275
Reducing the burden	26	16.05	136	83.95	68.31	13.65	64.90	14.01	-1.140	.256		-.244
Improving the environment	25	15.43	137	84.57	67.48	13.50	65.08	14.06	-0.789	.431		-.172
Support for the implementation of PA (non-interpersonal)	6	3.70	156	96.30	67.33	7.97	65.38	14.15	-0.336	.738		-.140
Support for the implementation of PA (interpersonal)	27	16.67	135	83.33	72.22	11.55	64.10	14.05	-2.819	.005	**	-.594
Evaluating the implementation of PA	2	1.23	160	98.77	75.50	7.78	65.33	13.99	-1.024	.307		-.729
Support for improvement of physical condition	14	8.64	148	91.36	66.79	15.00	65.32	13.91	-0.373	.709		-.104
Difficulties due to social situations and physical conditions	20	12.35	142	87.65	62.30	14.37	65.89	13.90	1.078	.283		.258
None	50	30.86	112	69.14	62.18	13.97	66.91	13.77	2.011	.046	*	.342

** $p < .01$. * $p < .05$
